# First Detection and Phylogenetic Analysis of Equine Hepacivirus (EqHV) in Iran

**DOI:** 10.1002/vms3.70737

**Published:** 2025-12-20

**Authors:** Mahdi Pourmahdi Borujeni, Hamzeh Ghobadian Diali, Alireza Ghadrdan Mashhadi, Mahdi Jalali Aliabad

**Affiliations:** ^1^ Department of Food Hygiene, Faculty of Veterinary Medicine Shahid Chamran University of Ahvaz Ahvaz Iran; ^2^ Department of Pathobiology, Faculty of Veterinary Medicine Shahid Chamran University of Ahvaz Ahvaz Iran; ^3^ Department of Clinical Sciences, Faculty of Veterinary Medicine Shahid Chamran University of Ahvaz Ahvaz Iran

**Keywords:** *Hepacivirus equi*, Iran, phylogenetic, PQ848112

## Abstract

**Background:**

The recent identification of novel viruses associated with hepatitis in horses has prompted equine veterinarians to investigate the viral factors contributing to equine hepatitis. *Hepacivirus equi* (*EqHV*), a member of the *Flaviviridae* family within the *Hepacivirus* genus, has been detected in horses affected by hepatitis. Globally, *EqHV* is highly conserved, existing as a single genotype with three distinct subtypes (Subtypes 1–3). Numerous studies have detected the virus by PCR and identified EqHV‐specific antibodies through serological tests in different regions worldwide. However, to the best of our knowledge, no published research has investigated the presence of *EqHV* in the horse population of Iran.

**Objectives:**

This study aimed to investigate the presence of *Hepacivirus equi* in Iran's horse population and to perform a phylogenetic analysis of the detected strains.

**Methods:**

A total of 150 whole blood samples were collected from horses (*Equus caballus*) across various locations in Khuzestan Province, Iran. Molecular assays were used to detect the EqHV genetic material.

**Results:**

This study confirms the presence of *EqHV* in the equine population of Khuzestan, Iran, with an average prevalence of 4.66%. Phylogenetic analysis revealed that the strain identified in this study, designated as ‘IR1‐Ahvaz‐2024’, belongs to *EqHV‐1* subtype. The sequence identified in this study has been submitted to GenBank under accession number PQ848112.

**Conclusion:**

The detection of *EqHV* in Iran's horse population is of potential significance to manufacturers and users of equine‐derived biological products, both in Iran and in countries that import these products. These findings highlight the need for further research and surveillance to assess the potential impact of *EqHV* on equine health in the region.

## Graphical abstract

This study presents the first detection *of Hepacivirus equi (EqHV)* in the equine population of Khuzestan Province, Iran. Molecular analysis revealed a prevalence rate of 4.66%, while phylogenetic analysis confirmed that the strain IR1‐Ahvaz‐2024 belongs to *EqHV* subtype 1.

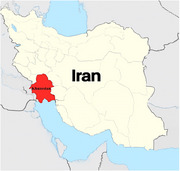

## 1 Introduction

Equines play a vital role in pharmaceutical manufacturing and veterinary medicine, particularly as donors for the production of critical products such as antisera and antivenoms. Consequently, the health status of these animals directly impacts the safety and quality of derived products, as well as the reliability of veterinary interventions. In recent years*, Hepacivirus equi* (*EqHV*) has garnered increasing attention due to its ability to establish subclinical infections and its potential implications for the biosafety of equine‐derived biological materials (Pfaender, Walter, et al. 2015; Tomlinson et al. 2019). These concerns are especially relevant in regions where horse‐derived biologics are widely produced and utilized.

*EqHV* belongs to the *Flaviviridae* family (Simmonds et al. 2017) and is classified under the *Hepacivirus* genus. It was first identified by Kapoor and colleagues in the United States in 2011. Genomic studies have shown that *EqHV* is highly conserved, consisting of a single genotype with three distinct subtypes (1–3), which are globally distributed without geographic restrictions (Lu et al. 2019; Pacchiarotti et al. 2022; Wu et al. 2020). Notably, *EqHV* is considered the closest known genetic relative of the human hepatitis C virus (HCV) (Burbelo et al. 2012), positioning *EqHV* as a valuable surrogate model for studying hepaciviral pathogenesis.

Multiple studies have demonstrated that EqHV exhibits hepatotropism, with viral RNA and histological evidence confirming liver involvement (Pfaender, Cavalleri, et al. 2015; Ramsay et al. 2015; Scheel et al. 2015). Although the precise natural transmission routes of *EqHV* remain unknown, experimental and clinical findings have shown that it can be transmitted via blood or serum through iatrogenic means (Pacchiarotti et al. 2022; Pfaender et al. 2017; Ramsay et al. 2015). Additionally, Gather et al. (2016) detected *EqHV* RNA in the umbilical cord blood and serum of a foal born to an infected mare, suggesting that vertical transmission may also occur. To date, no additional definitive routes of *EqHV* transmission or shedding have been identified beyond those previously described. *EqHV* infections have been reported to persist for several months and, in some cases, may progress to chronic infection (Pfaender et al. 2017; Ramsay et al. 2015; Scheel et al. 2015). Given the asymptomatic nature of most infections, detecting EqHV requires sensitive molecular and serological techniques (Pfaender et al. 2017; Ramsay et al. 2015).

Global surveillance studies have indicated varying levels of *EqHV* prevalence, with RNA detection rates ranging from 2% to 18% and seroprevalence rates between 22% and 84% across different regions (Pacchiarotti et al. 2022). Such variability underscores the importance of regional monitoring, particularly in countries involved in the manufacture or export of equine‐derived biological products. Surveillance of *EqHV* prevalence is essential not only for safeguarding animal health and ensuring the safety of biological products but also for maintaining biosafety standards and preserving confidence in international trade (Divers et al. 2018; Gather et al. 2016; Pfaender, Walter, et al. 2015b; Tomlinson et al. 2019). To the best of our knowledge, no published information currently exists regarding the investigation of *EqHV* in the horse population of Iran. Accordingly, this study represents the first molecular investigation of *EqHV* in Iran's equine population.

## 2 Materials and Methods

### 2.1 Study Design and Sampling

This study was conducted in Khuzestan Province, located in the southwestern region of Iran. Geographically, the province lies between 29°58′ N and 33°04′ N latitude and 47°41′ E and 50°39′ E longitude, according to the WGS 84 geographic coordinate system (EPSG:4326). It shares a border with Iraq to the west and the Persian Gulf to the south. Khuzestan was selected as the target region for this molecular investigation due to its strategic location, abundant natural resources and well‐established equine industry. A two‐stage cluster sampling approach was employed to select study sites. In the first stage, five cities—Ahvaz (POINT [48.6833, 31.3167]), Mahshahr (POINT [49.1833, 30.5500]), Ramhormoz (POINT [49.6000, 31.2667]), Susangerd (POINT [48.1667, 31.5500]) and Shushtar (POINT [48.4558 31.2755])—were randomly chosen to represent diverse geographic and epidemiological zones within the province. In the second stage, horses were randomly selected within each city. The required sample size for each city was calculated using a detection‐based formula to ensure, with 95% confidence, the detection of at least one *EqHV*‐positive animal, assuming a minimum expected prevalence of 10%, as supported by previous literature (Chen et al. 2021; Lu et al. 2016; Matsuu et al. 2015; Yoon et al. 2022). The formula used was: *n* = [1 − (1 − P)^(1/^ *^d^* ^)^] × [(*N* − d)/2] + 1 where: *n* = required sample size per city, *p* = probability of detecting at least one positive animal (set at 0.95), *N* = total equine population in each city and *d* = expected number of positive animals (10% of *N*) (Thrusfield et al. 2018).

Based on equine population data and these parameters, the required sample size ranged from 21 to 29 horses per city. In total, 150 horses (*Equus caballus*) were randomly sampled from the four selected cities between October and December 2024 (Table 1) for molecular detection and phylogenetic analysis of *EqHV*. This study involved the collection of blood samples from healthy Arabian racehorses. All procedures were conducted in full compliance with the ethical guidelines outlined in Ethical Code No. 981060218, issued by the Faculty of Veterinary Medicine at Shahid Chamran University of Ahvaz, with strict adherence to animal welfare principles throughout the research process. Prior to blood collection, the health status of each horse was thoroughly assessed and measures were implemented to minimize any potential discomfort or distress. Furthermore, permission was obtained from the horse owners before proceeding with the procedure. Blood collection was carried out by trained veterinary professionals, with all necessary precautions taken to ensure the safety of both the animals and the researchers.

**TABLE 1:** Detailed information regarding the collection period, geographic origin and the number of equine samples used for molecular research on *EqHV* in Iran during 2024.

Collection period	Geographic origin	Number
May, 2024	Ahvaz	50
October, 2024	Mahshahr	25
October, 2024	Ramhormoz	25
November, 2024	Susangerd	25
November, 2024	Shushtar	25
Total		150

### 2.2 RNA Extraction, cDNA Synthesis and PCR Amplification

Molecular assays were conducted to detect *EqHV* RNA and perform subsequent genomic characterization. Total RNA was extracted from each whole blood sample using the SinaPure RNA Kit (SinaClon, Iran). cDNA synthesis was carried out with the SinaClone First Strand cDNA Synthesis Kit (SinaClon, Iran). To amplify a target region in the 5′ UTR of the *EqHV* genome, two sets of degenerate primers from Lu et al. (2016) were used. To enhance phylogenetic resolution, an additional PCR step was performed on *EqHV*‐positive samples, based on the method by Lu et al. (2016), using a primer pair specifically designed for this study. The goal of this step was to generate an extended amplicon from the 5′ UTR region of EqHV, as outlined in Table 2. For this purpose, the complete genome sequence of *EqHV* was downloaded from GenBank (JPN3/JAPAN/2013 [NC_024889]). Specific primers were manually designed and analyzed using the Oligo 6.31 program (Molecular Biology Insights, Inc., CO), targeting genomic sequences spanning from the 5′ UTR (nucleotide positions 18–37) to the core protein C gene (nucleotide positions 428–447).

**TABLE 2:** Primer sequences used in this study.

Primer name	Primer sequence (primer sense: 5′ to 3′)	Product size (bp)	Reference
NS5UO‐F	ACAYYACCATGTGTCACTCCCCCT	≈310	Lu et al. (2016)
NS5UO‐R	CYCATGTCCTATGGTCTACGAGA		
NS5UI‐F	ACACGGAAAYRRGTTAACCAYACYC	230	Lu et al. (2016)
NS5UI‐R	GCCCTCGCAAGCATCCTATCAG		
5'UTR‐F	GGTGCGTTGTCAGCGTTTTG	430	This study
5'UTR‐R	CCCTATTCCGTGGTCCTCTT		

The conventional nested PCR (producing 310 and 230 base pair [bp] fragments) and conventional PCR (producing a 430 bp fragment) cycling profile for all primer sets and rounds consisted of an initial denaturation at 95°C for 5 min, followed by 40 cycles of 95°C for 1 min, 60°C for 30 s and 72°C for 30 s, concluding with a final extension at 72°C for 3 min. Each PCR reaction contained 12.5 µL of 2× Amplicon master mix (Denmark), 2 µL of template cDNA/PCR product, 1 µL of each primer (final concentration: 10 pmol) and nuclease‐free water to achieve a final volume of 25 µL. Finally, the PCR products were subjected to electrophoresis on a 1% agarose gel.

### 2.3 Sequencing and Phylogenetic Analysis

A 430 bp PCR amplicon produced with primers that target the 5′ UTR region was sent to Gene Fanavaran Company (Iran) for Sanger dideoxy sequencing along with the corresponding primer pairs. The raw sequencing data were manually edited using BioEdit software (version 7.2.5.0). A Basic Local Alignment Search Tool (BLAST) analysis was performed to verify the accuracy of the obtained nucleotide sequence. For phylogenetic reconstruction, 35 species were selected, including all known subtypes of *EqHV* (Pacchiarotti et al. 2022) and rodent *Hepacivirus* (NC_021153) as an outgroup species. Sequences were aligned using MAFFT software version 7.526, with manual adjustments (Katoh and Standley 2013). Phylogenetic analysis was conducted using both Bayesian inference and maximum likelihood (ML) approaches.

#### 2.3.1 Bayesian Phylogenetic Analysis

The optimal model of sequence evolution was determined using MrModeltest version 2.3 (JAA) based on the Akaike Information Criterion (AIC) (Posada and Buckley 2004), identifying GTR+I+G as the best‐fit model for nucleotide substitution. Bayesian phylogenetic analysis was performed using MrBayes version 3.1.2 (Ronquist and Huelsenbeck 2003). Model parameter posteriors were estimated from the data using default priors. The analysis was conducted over 10 million generations using the Markov Chain Monte Carlo (MCMC) method. Two independent runs (*N*_runs_ = 2) were initiated from distinct random trees, each consisting of four Markov chains, with trees sampled every 100 generations. The first 25% of trees were discarded as the burn‐in period, and the remaining trees were used to construct a consensus tree based on a 50% majority rule, supported by posterior probability (PP) values. Tree visualization was performed using TreeGraph 2 version 2.15.0‐887 beta (Stöver and Müller 2010) (Available at: http://treegraph.bioinfweb.info/Download).

#### 2.3.2 ML Phylogenetic Analysis

ML analysis was conducted using the CIPRES Science Gateway with RAxML HPC v.8 on XSEDE. The GTRGAMMA model with the −Fa parameter was applied for rapid bootstrapping and optimal ML tree search, with 1000 bootstrap replicates. Default settings were used for all other parameters. Available at: https://phylo.org/.

## 3 Results

### 3.1 PCR Results

In this study, samples displaying visible bands at 310 or 230 base pairs were considered positive. Accordingly, 7 (4.66%) of the 150 equine whole blood samples examined had positive *EqHV* RNA results. The distribution of positive cases by region was as follows: Ahvaz: 5 out of 50 samples (10.0%), Mahshahr: 1 out of 25 samples (4.0%), Ramhormoz: 0 out of 25 samples (0.0%), Susangerd: 1 out of 25 samples (4.0%) and Shushtar: 0 out of 25 samples (0.0%). Among the seven positive samples, only one (from Ahvaz) produced a clear and reliable band suitable for sequencing; therefore, it was selected for subsequent phylogenetic analysis.

### 3.2 Sequencing and Phylogenetic Results

Sequence analysis of the PCR amplicon, combined with an initial BLAST analysis, confirmed a high degree of sequence homology between the examined strain (designated IR1‐Ahvaz‐2024) and previously identified *EqHV* isolates. Phylogenetic analysis further validated the classification of IR1‐Ahvaz‐2024 as *EqHV*, leading to its official registration in GenBank under accession number PQ848112 for public access.

**FIGURE 1:**
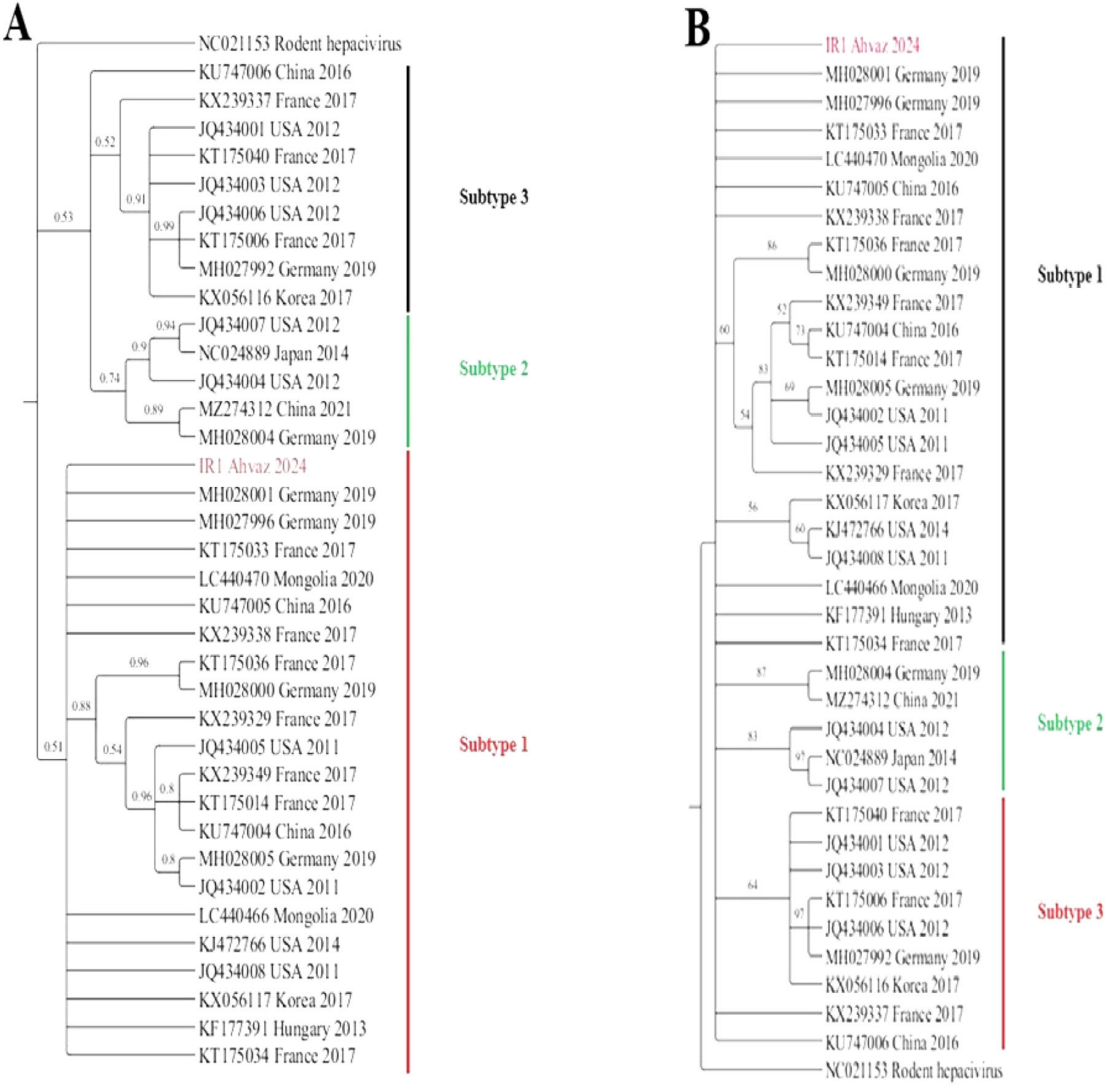
Phylogenetic analysis of *EqHV* based on the 5ʹ UTR region using Bayesian inference (A) and Maximum Likelihood (B) methods. Rodent *Hepacivirus* (NC 021153) was selected as an outgroup for phylogenetic tree reconstruction among *EqHV* species. The analysis confirmed that IR1‐Ahvaz‐2024, identified in this study, clusters within *EqHV* subtype 1 (highlighted in red).

Phylogenetic analysis of *EqHV*, based on the 5ʹ UTR region, was conducted using both ML and Bayesian inference methods. The results obtained from both approaches were highly consistent (Figure 1A,B). Our findings confirm that IR1‐Ahvaz‐2024 belongs to *EqHV* Subtype 1. Although distinct sister clades were identified across all subtypes, the phylogenetic relationships among certain species within these subtypes remain unresolved. For further details, refer to Figure 1.

## 4 Discussion

To investigate the presence of *EqHV* in Iran, 150 whole blood samples were collected from horses in urban and rural areas of Khuzestan province. These samples were analyzed using RT‐Nested PCR for *EqHV* detection. This study presents the first molecular confirmation of *EqHV* in Iran, with a low but notable prevalence. A systematic review and meta‐analysis conducted by Pacchiarotti et al. (2022) reported the average molecular prevalence of *EqHV* as follows: 6.2% in China, 13.7% in Japan, 8.1% in South Korea, 11.2% in Australia, 6.96% in Germany, 5.9% in France, 7.9% in South Africa, 10.5% in Morocco, 10.99% in the United States, 12% in Brazil and 4.7% in Italy. Based on this data, the prevalence of *EqHV* in Iran's horse population is lower than that in the countries listed above. However, Pacchiarotti et al. (2022) also found that the molecular prevalence of *EqHV* is 1.27% in the United Kingdom and 2.26% in Austria, which are lower than the prevalence observed in our study.

*EqHV* is highly genetically conserved, with a single known genotype and three subtypes (Subtypes 1–3) that are distributed worldwide without a clear geographical preference (Lu et al. 2019; Wu et al. 2020). However, the classification of *EqHV* subtypes remains a subject of debate. While the term ‘subtype’ conventionally denotes antigenic variation, in the case of *EqHV*, this classification is based solely on sequence divergence, without confirmed antigenic or functional differences among the subtypes. Therefore, further research is warranted to validate the robustness and biological relevance of this proposed classification. Nonetheless, based on Lu et al.’s classification system, our phylogenetic analysis identified the detected virus as *EqHV* Subtype 1 (Figure 1).

The precise statistical power could not be determined because this study was exploratory. Instead of relying on precise statistical methods, we adopted a detection‐based approach to increase the likelihood of detecting at least one animal infected with EqHV. As a result, the reported prevalence might not accurately reflect the true prevalence of EqHV in the equine population of Iran. Because the study was limited to Khuzestan Province, broader investigations in other regions of Iran are needed to obtain a more comprehensive understanding of *EqHV* distribution nationwide. In addition, we recommend full‐genome sequencing in future research to better characterize the genetic diversity and evolutionary dynamics of *EqHV* in the Iranian equine population. These initiatives would improve worldwide understanding of *EqHV* in addition to enhancing local viral surveillance and control. Despite these issues, this study is the first to identify *EqHV* in Iran, and the findings significantly increase our knowledge of the virus's prevalence in the nation. It also provides important information that will guide future epidemiological and phylogenetic studies.

Although *EqHV* infections are often subclinical and asymptomatic (Burbelo et al. 2012; Ramsay et al. 2015), their detection in equine populations poses significant concerns for biosafety and public health. The equine biological product sector, dependent on serum, plasma and other materials sourced from equine donors, is significantly affected by the identification of *EqHV* in horses. The utilization of *EqHV*‐positive animals for the production of biologics, including antitoxins, vaccines and immunoglobulins, raises concerns about the virological safety and regulatory compliance of these products, especially in international markets with stringent biosafety regulations (Pfaender, Walter, et al. 2015b; Tomlinson et al. 2019).

While investigations into *EqHV*’s direct pathogenicity in equines continue, the virus's genetic resemblance to HCV, its persistence and vertical transmission (Gather et al. 2016) underscore the necessity for enhanced molecular surveillance and risk evaluation. Therefore, *EqHV* should be considered a potential biosafety risk in veterinary and pharmaceutical manufacturing, in addition to its proposed therapeutic application in veterinary medicine. To ensure the purity, safety and international acceptance of equine‐derived biological products, it is recommended that horse donors undergo routine screening for *EqHV*. Considering that Iran manufactures biological products sourced from equines, our findings hold practical significance for both manufacturers and consumers, emphasizing the importance of routine screening for *EqHV* to ensure the safety of equine‐derived products.

## Author Contributions

**Mahdi Pourmahdi Borujeni**: methodology, project administration, supervision, resources, funding acquisition, validation. **Hamzeh Ghobadian Diali**: writing – original draft, software, validation, visualization, writing – review and editing, data curation, resources, conceptualization. **Alireza Ghadrdan Mashhadi**: writing – review and editing, investigation, validation. **Mahdi Jalali Aliabad**: formal analysis, data curation, software.

## Ethics Statement

This study, which involved blood collection from horses, was conducted in strict accordance with the ethical guidelines set forth in Ethical Code No. 981060218 of the Faculty of Veterinary Medicine at Shahid Chamran University of Ahvaz. All procedures were performed with the utmost respect for ethical principles, ensuring the welfare and well‐being of the animals throughout the research process. Prior to blood collection, the health status of each horse was thoroughly assessed and measures were implemented to minimize any potential discomfort or distress. Furthermore, permission was obtained from the horse owners before proceeding with the procedure. The blood collection was carried out by trained veterinary professionals, with all necessary precautions taken to ensure the safety of both the animals and the researchers.

## Conflicts of Interest

The authors declare no conflicts of interest.

## Data Availability

The data supporting the findings of this study are included within the article.
